# Lessons from the Tōhoku tsunami: A model for island avifauna conservation prioritization

**DOI:** 10.1002/ece3.3092

**Published:** 2017-06-22

**Authors:** Michelle H. Reynolds, Paul Berkowitz, John L. Klavitter, Karen N. Courtot

**Affiliations:** ^1^ U.S. Geological Survey Pacific Island Ecosystems Research Center Hawai'i National Park HI USA; ^2^ Hawai'i Cooperative Studies Unit University of Hawai'i at Hilo Hawai'i National Park HI USA; ^3^ U.S. Fish and Wildlife Service National Wildlife Refuge System Falls Church VA USA

**Keywords:** Laysan Island, Midway Atoll, Papahanaumokuakea, sea‐level rise, seabirds, seismic sea wave

## Abstract

Earthquake‐generated tsunamis threaten coastal areas and low‐lying islands with sudden flooding. Although human hazards and infrastructure damage have been well documented for tsunamis in recent decades, the effects on wildlife communities rarely have been quantified. We describe a tsunami that hit the world's largest remaining tropical seabird rookery and estimate the effects of sudden flooding on 23 bird species nesting on Pacific islands more than 3,800 km from the epicenter. We used global positioning systems, tide gauge data, and satellite imagery to quantify characteristics of the Tōhoku earthquake‐generated tsunami (11 March 2011) and its inundation extent across four Hawaiian Islands. We estimated short‐term effects of sudden flooding to bird communities using spatially explicit data from Midway Atoll and Laysan Island, Hawai'i. We describe variation in species vulnerability based on breeding phenology, nesting habitat, and life history traits. The tsunami inundated 21%–100% of each island's area at Midway Atoll and Laysan Island. Procellariformes (albatrosses and petrels) chick and egg losses exceeded 258,500 at Midway Atoll while albatross chick losses at Laysan Island exceeded 21,400. The tsunami struck at night and during the peak of nesting for 14 colonial seabird species. Strongly philopatric Procellariformes were vulnerable to the tsunami. Nonmigratory, endemic, endangered Laysan Teal (*Anas laysanensis*) were sensitive to ecosystem effects such as habitat changes and carcass‐initiated epizootics of avian botulism, and its populations declined approximately 40% on both atolls post‐tsunami. Catastrophic flooding of Pacific islands occurs periodically not only from tsunamis, but also from storm surge and rainfall; with sea‐level rise, the frequency of sudden flooding events will likely increase. As invasive predators occupy habitat on higher elevation Hawaiian Islands and globally important avian populations are concentrated on low‐lying islands, additional conservation strategies may be warranted to increase resilience of island biodiversity encountering tsunamis and rising sea levels.

## 1 INTRODUCTION

Seismic events have produced hazardous tsunamis that affect coastlines, damage infrastructure, and cause human causalities approximately once per year globally (Bernard, 2001). The Hawaiian Archipelago is vulnerable to tsunamis generated in most parts of the Pacific Basin (NOAA 2017). Depending on water depth, tsunamis can propagate from the epicenter at speeds of greater than 1,000 km/hr (NOAA, 2017) refracting near islands and bays, so their impacts vary greatly between locations. Little is known about tsunamis on remote islands, and the effects of sudden flooding on island biodiversity are not well studied. After the 26 December 2004 Indian Ocean tsunami following the Sumatra‐Andaman earthquake (Mw = 9.1; max water height near source 50.9 m; NOAA, 2015b), researchers documented effects to wildlife and ecosystems including King Penguins (*Aptenodytes patagonicus*) in the Crozet Archipelago (Viera, Le Bohec, Cote, & Groscolas, 2006); Nicobar Megapodes (*Megapodius nicobariensis*; Sivakumar, 2009), and mangrove, coral reef, and forest ecosystems (Ramachandran et al., 2005) of the Nicobar Islands; and plant communities of Phuket Island (Hayasaka, Goka, Thawatchai, & Fujiwara, 2012).

**Figure 1:**
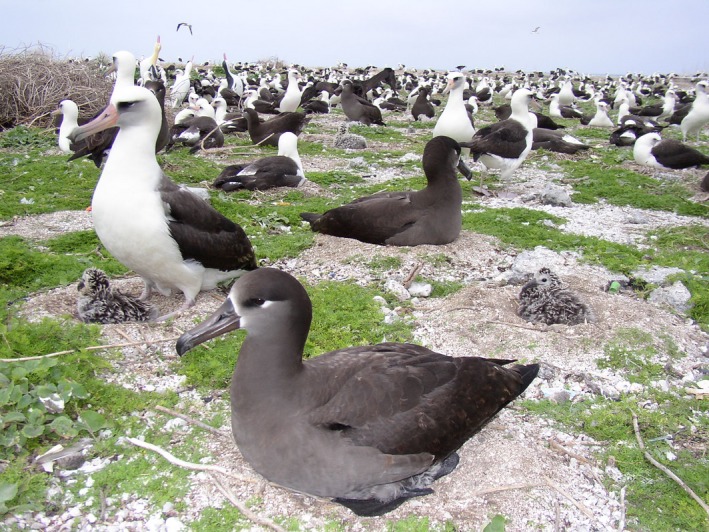
Black‐footed (*Phoebastria nigripes*) and Laysan albatrosses (*Phoebastria immutabilis*) attending nests at Midway Atoll, Hawai'i

Many island species have limited global distributions and consequently remain highly vulnerable to catastrophic disturbances such as tsunamis, high surf, hurricanes, and volcanic eruptions (Finkelstein et al., 2010; Hahn, Vergara, Baumeister, Soto, & Römer, 2015; Porwal, Padalia, & Roy, 2012; Reynolds, Courtot, Berkowitz, Storlazzi, & Flint, 2015; Reynolds, Courtot, Brinck, Rehkemper, & Hatfield, 2015; Underwood, 2013). Since human colonization and introduction of mammalian predators, many Pacific seabird colonies and endemic land bird populations were extirpated from islands across their range and today are anthropogenically restricted and often limited to small protected and predator‐free islands (Burney et al., 2001; Olson & James, 1982; Rauzon, 2001; Steadman, 2006). Twenty‐one seabird and three Hawai'i endemic land bird species breed at Midway Atoll and/or Laysan Island (Reynolds, Berkowitz, Courtot, & Krause, 2012). In this study, we mapped tsunami‐driven habitat inundation and estimated the impacts to breeding bird communities. We quantified flooding extent, summarized tide gauge data (NOAA 2014), and estimated habitat or nest losses for 10 species with empirical distribution or abundance data: Black‐footed Albatross (*Phoebastria nigripes*; Figure 1), Laysan Albatross (*P. immutabilis*), Bonin Petrel (*Pterodroma hypoleuca*), Masked Booby (*Sula dactylatra*), Brown Booby (*S*.* leucogaster*), Red‐footed Booby (*S*.* sula*), Great Frigatebird (*Fregata minor*), and endangered Short‐tailed Albatross (*P. albatrus*), Laysan Teal (*Anas laysanensis*), and Laysan Finch (*Telespiza cantans*). For 13 additional species without population monitoring (Table 1), we describe habitat inundation, habitat vulnerability, and the expected impacts from the Tōhoku tsunami.

**Table 1:** Nesting habitat used by birds at Midway Atoll (M) and Laysan Island (L), Hawai'i

Nesting habitat	Species	Nesting Island
Tree and shrub canopy (C)	White‐tailed Tropicbird (*Phaethon lepturus*)	M
	Red‐footed Booby (*Sula sula*)	L, M
	Great Frigatebird (*Fregata minor*)	L, M
	Black Noddy (*Anous minutus*)	L, M
	White Tern (*Gygis alba*)	L, M
	Laysan Finch (*Telespiza cantans*)	L
Underneath trees, shrubs and dense bunch grasses on the ground (U)	Christmas Shearwater (*Puffinus nativitatis*)	L, M
	Red‐tailed Tropicbird (*Phaethon rubricauda*)	L, M
	Laysan Teal (*Anas laysanesis*)	L, M
On the ground with vegetation or bare ground (G)	Black‐footed Albatross (*Phoebastria nigripes*)	L, M
	Laysan Albatross (*Phoebastria immutabilis*)	L, M
	Short‐tailed Albatross (*Phoebastria albatrus*)	M
	Masked Booby (*Sula dactylatra)*	L, M
	Brown Booby (*Sula leucogaster*)	L, M
	Gray‐backed Tern (*Onychoprion lunatus*)	L, M
	Sooty Tern (*O*. *fuscatus*)	L, M
	Little Tern (*Sternula albifrons*)	M
	Least Tern (*Sternula antillarum)*	M
	Brown Noddy (*Anous stolidus*)	L, M
Subterranean burrows and crevices (S)	Bonin Petrel (*Pterodroma hypoleuca*)	L, M
	Bulwer's Petrel (*Bulweria bulwerii*)	L^a^
	Wedge‐tailed Shearwater (*Ardenna pacifica*)	L, M
	Tristram's Storm‐petrel (*Oceanodroma tristrami*)	L^b^

a Lays eggs in artificial burrows at Midway Atoll, but fledging not confirmed.

b Nesting and fledging confirmed at Midway Atoll in 2016.

## 2 METHODS

**Figure 2:**
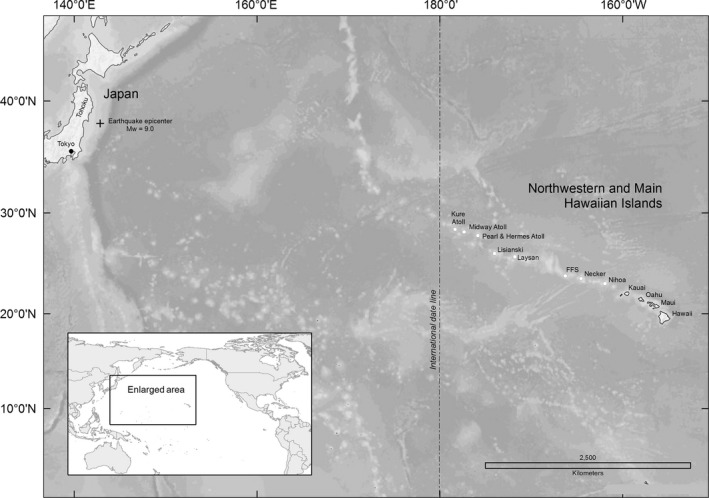
Map of the 11 March 2011 earthquake epicenter in relation to the Northwestern and main Hawaiian Islands

A series of islands, atolls, and seamounts located in the subtropical Central Pacific Ocean are a part of the largest conservation area in the United States, Papahānaumokuākea Marine National Monument (PMNM; Presidential Proclamation 2016). The coral atoll of Midway (28°11′41″–28°16′50″ N and 177°18′38″–177°25′38″ W) lies approximately 2,300 km from Honolulu and consists of three islands: Sand (457.7 ha; mean elevation 3.2 m), Spit (5.1 ha; mean elevation 1.5 m), and Eastern (136.4 ha; mean elevation 2.6 m; Figure 2). Laysan Island (25°46′11″ N and 171°44′00″ W) covers 412.0 ha (including a hypersaline lake) and has a mean elevation of 3.9 m (Reynolds et al., 2012).

At 5:46 UTC (14:46 Japan Standard Time) on 11 March 2011, a magnitude 9.0 tsunamigenic earthquake occurred off the Tōhoku coast of Japan (NOAA 2017; Figure 2). The associated waves propagated across the Pacific. The source area of the tsunami (approximately 500 km long and 200 km wide) was along the western edge of the Japanese Trench (Hayashi, Tsushima, Hirata, Kimura, & Maeda, 2011; Maeda, Furumura, Sakai, & Shinohara, 2011). Data showed a maximum inundation height (height of wave at the coastline) of over 19 m, a maximum run‐up elevation (highest terrestrial elevation reached by the wave) of 39.7 m (Mimura, Yasuhara, Kawagoe, Yokiki, & Kazama, 2011), and run‐up distance (inland inundation distance) extending more than 5 km inland of Japan's east coast (Mori, Takahashi, Yasuda, & Yanagisawa, 2011).

### 2.1 Tide station data

To characterize the water levels around the Hawaiian archipelago, we extracted unprocessed data from the tide gauge at Sand Island and used 1‐min intervals from 11 to 13 March 2011 (Station 1619910; NOAA 2014). We compared the 1‐minute water levels with 15‐second data to verify that a smaller time increment was not needed. We also extracted data from Kahului Harbor, Maui (Station 1615680) and across the Pacific at Crescent City, California (Station 9419750; NOAA 2015).

### 2.2 Tsunami inundation

The line of tsunami debris was traversed with global positioning system (GPS) units on 15 March 2011 at Midway Atoll (Trimble GeoXT GPS unit with <1 m accuracy) and 12 March 2011 at Laysan Island (Garmin units GPS76CSX or Garmin 530HCx with <5 m accuracy; USFWS unpublished data). We uploaded these GPS data into ArcGIS 10 (Environmental Systems Research Institute, 2010) and created inundation polygons for the area between the coastline and inundation line. Mean sea level (MSL; i.e., coastline) for Midway Atoll was delineated based on satellite imagery (collected on 14 January 2010; DigitalGlobe Inc., 2010) and verified tide, as measured at NOAA Station 1619910 (NOAA, 2011). The coastline of Laysan Island was digitized using WorldView‐2 satellite imagery (collected 18 May 2010; DigitalGlobe Inc., 2010) with the predicted tides for MSL (Berkowitz, Storlazzi, Courtot, Krause, & Reynolds, 2012). To calculate wave run‐up elevations, we used recent digital elevation models (DEMs; PhotoSat Information Ltd., 2010, 2011). To assess how sudden flooding impacted island bird communities, we overlaid the tsunami inundation area with species‐specific population distributions and land cover/habitat data.

### 2.3 Habitat mapping

We estimated nesting habitat of breeding bird communities by generalizing the land cover classification from satellite imagery into four nest substrates or sites: (1) in trees and shrubs, (2) underneath the trees, shrubs, and dense grasses, (3) on the ground, or (4) below ground in subterranean burrows and crevices (Table 1). We classified the land cover as described in Reynolds et al. (2012) using WorldView‐2 satellite imagery (DigitalGlobe Inc., 2010) of Midway Atoll collected 14 January 2010 and of Laysan Island collected on 18 May 2010 (DigitalGlobe Inc., 2010).

### 2.4 Bird distributions and abundance

**Figure 3:**
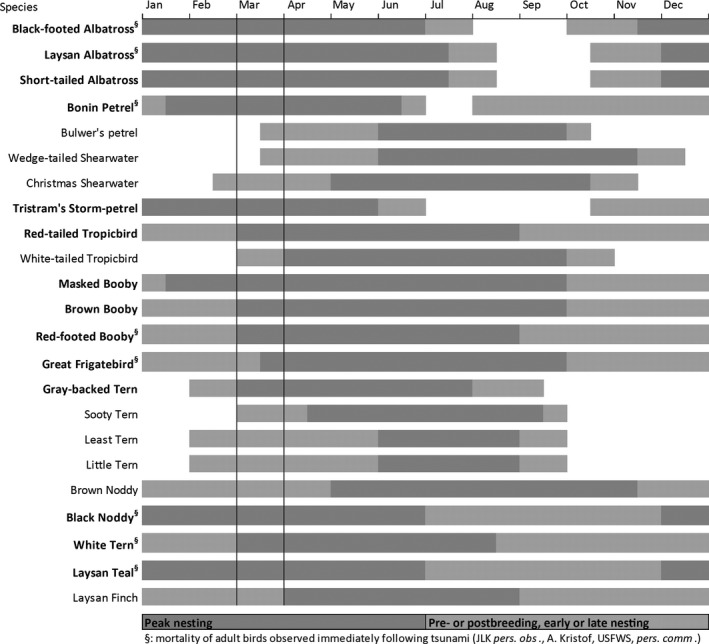
Breeding phenology of 23 bird species breeding at Midway Atoll and/or Laysan Island, Hawai'i. Species indicated in bold have peak breeding seasons that coincided with the March 2011 tsunami

To estimate impacts to birds, we compiled nesting phenology, habitat use, distribution, and abundance data (Tables 1 and 2; Figure 3; for details see Moore, 2009; Reynolds et al., 2012; Reynolds, Courtot, Brinck et al., 2015; Reynolds, Courtot, Berkowitz et al., 2015). Data collected prior to the tsunami included breeding distribution and/or abundance data and land cover imagery for habitat delineation of Black‐footed Albatross, Laysan Albatross, Short‐tailed Albatross, Bonin Petrel, and Laysan Teal at Midway Atoll and Black‐footed Albatross, Laysan Albatross, Red‐footed Booby, Great Frigatebird, Masked Booby, Brown Booby, Laysan Teal and Laysan Finch at Laysan Island (Table 2). After the tsunami, we conducted a spatially explicit census of Black‐footed and Laysan albatross nests and collected GPS locations for 92% of the Black‐footed Albatross nests within the tsunami inundation area (Trimble GeoXM and GeoXT units; ± <3 m accuracy); additional nest site locations were estimated from atoll‐wide census data (see Reynolds, Courtot, Berkowitz et al., 2015; USFWS unpublished data). The albatross census was conducted December 2011–January 2012 (USFWS unpublished data). Long‐term monitoring data (systematic bi‐monthly surveys; USFWS unpublished data) of Laysan Teal were analyzed to estimate population changes following the tsunami (Reynolds, Brinck, & Laniawe, 2011; Reynolds, Courtot, Brinck et al., 2015). Other species are not monitored for abundance, but their breeding phenology and habitat use is described to infer populations' vulnerability (Table 1; Figure 3).

**Table 2:** Species nest data: locations, years, methods, and sources applied to spatially explicit model of the effects of the Tōhoku earthquake‐generated tsunami at Midway Atoll and Laysan Island, Hawai'i, March 2011

Species	Location	Nesting year	Nest distribution data	Nest abundance data	Data and method sources
Black‐footed Albatross (*Phoebastria nigripes*)	Midway Atoll	2012	Subset of nest locations recorded (Trimble GeoXM and GeoXT, ± <3 m accuracy), all others: assumed equal distribution within spatially explicit census sectors within suitable habitat identified from land cover map	Direct count from atoll‐wide census	DigitalGlobe Inc., 2010; Reynolds, Courtot, Berkowitz et al., 2015; USFWS, unpublished data
	Laysan Island	2011	Transect grid, assumed equal distribution within suitable habitat identified from land cover map	Direct count from island‐wide census	Arata et al., 2009; DigitalGlobe Inc., 2010; Reynolds et al., 2012; USFWS, unpublished data
Laysan Albatross (*Phoebastria immutabilis*)	Midway Atoll	2011	Assumed equal distribution within spatially explicit census sectors within suitable habitat identified from land cover map	Direct count from atoll‐wide census	DigitalGlobe Inc., 2010; Reynolds, Courtot, Berkowitz et al., 2015; USFWS, unpublished data
	Laysan Island	2011	Transect grid, assumed equal distribution within suitable habitat identified from land cover map	Estimated from simple line transect	Buckland, Anderson, Burnham, & Laake, 1993; Arata et al., 2009; DigitalGlobe Inc., 2010; Reynolds et al., 2012; USFWS, unpublished data
Short‐tailed Albatross (*Phoebastria albatrus*)	Midway Atoll	2011	Location of single nest recorded during atoll‐wide census	Direct count from atoll‐wide census	USFWS, unpublished data
Bonin Petrel (*Pterodroma hypoleuca*)	Midway Atoll	2008	Nesting areas delineated by GPS (Garmin GPSMAP 60CSx, ± <10 m accuracy)	Estimated from burrow occupancy and density surveys	Moore, 2009; Reynolds, Courtot, Berkowitz et al., 2015
Masked Booby (*Sula dactylatra)*	Laysan Island	2009	Subset of nest locations recorded (Garmin GPSMAP 76, ± <10 m accuracy)	Partial count	Reynolds et al., 2012; USFWS, unpublished data
Brown Booby (*Sula leucogaster*)	Laysan Island	2009	Subset of nest locations recorded (Garmin GPSMAP 76, ± <10 m accuracy)	Partial count	Reynolds et al., 2012; USFWS, unpublished data
Red‐footed Booby (*Sula sula)*	Laysan Island	2008	Nesting area boundaries delineated (Garmin GPSMAP 76, ± <10 m accuracy)	Not estimated	Reynolds et al., 2012; USFWS, unpublished data
Great Frigatebird (*Fregata minor*)	Laysan Island	2008	Nesting area boundaries delineated (Garmin GPSMAP 76, ± <10 m accuracy)	Not estimated	Reynolds et al., 2012; USFWS, unpublished data
Laysan Teal (*Anas laysanensis*)	Midway Atoll	2009	Potential terrestrial nesting and foraging habitat quantified from land cover map	Not estimated^a^	DigitalGlobe Inc., 2010; Reynolds et al., 2011; USFWS & USGS^a^, unpublished data
	Laysan Island	2010	Potential terrestrial nesting and foraging habitat quantified from land cover map	Not estimated^a^	DigitalGlobe Inc., 2010; Reynolds et al., 2012; Reynolds, Courtot, Brinck et al., 2015, a; USFWS, unpublished data
Laysan Finch (*Telespiza cantans*)	Laysan Island	2010	Potential nesting and foraging habitat quantified from land cover map	Not estimated	DigitalGlobe Inc., 2010; Reynolds et al., 2012; USFWS, unpublished data

GPS, global positioning system; USFWS, US Fish and Wildlife Service; USGS, US Geological Survey.

a Population abundance estimated.

### 2.5 Uncertainty

Flooding extent uncertainty is primarily a function of the accuracy of the mapped inundation line (<5 m error) and species nest distributions. Nest sites were mapped as points (e.g., Masked and Brown Booby) while colony boundaries were mapped as linear features (e.g., Red‐footed Booby and Great Frigatebird), both accurate to within 5–10 m. For this model, we assumed the data reflected typical long‐term colony, distribution, and habitat for each species. Nest losses due to flooding were projected from empirical data of nesting distributions (Table 2) and were assumed to represent the nesting pattern at the time of the tsunami.

## 3 RESULTS

### 3.1 Inundation characteristics

**Figure 4:**
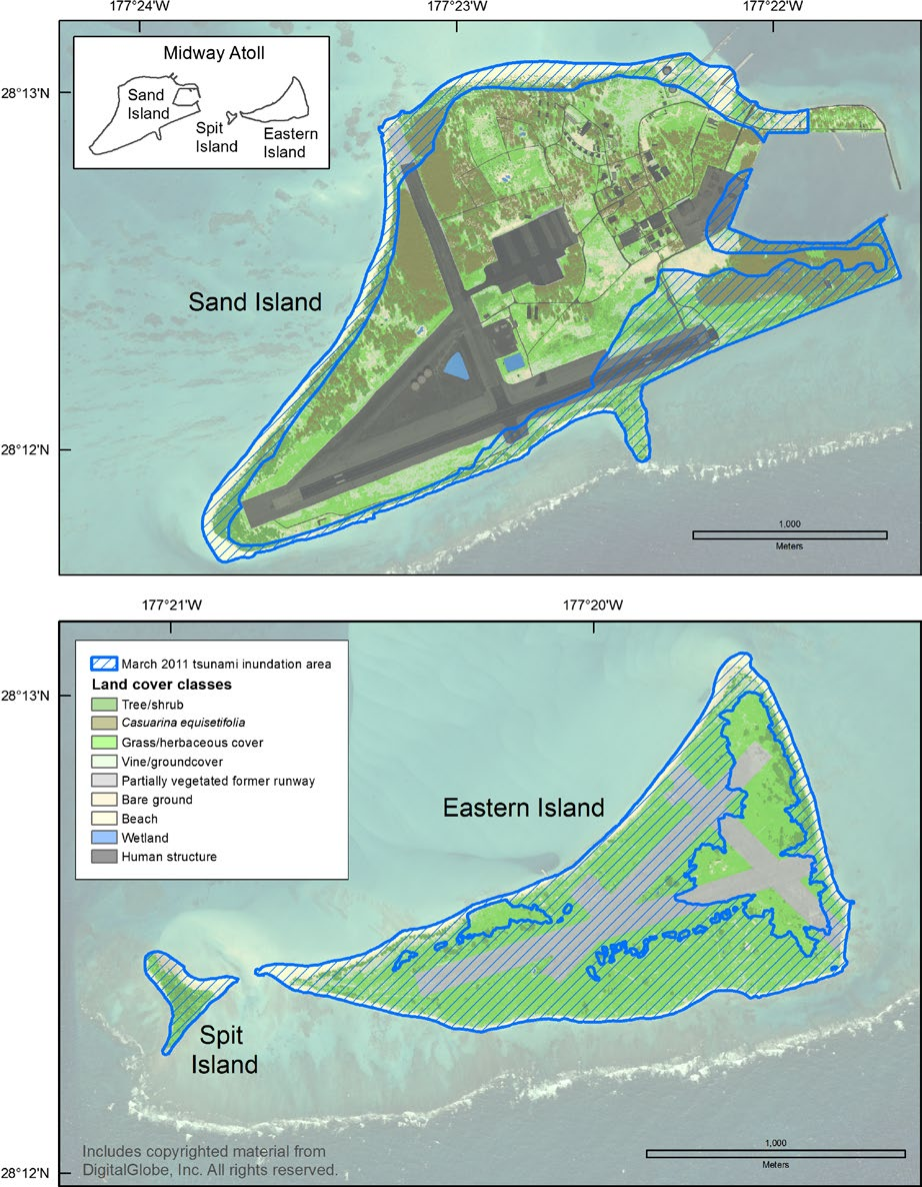
Inundation of land cover at Midway Atoll, Hawai'i during the March 2011 Tōhoku tsunami

**Figure 5:**
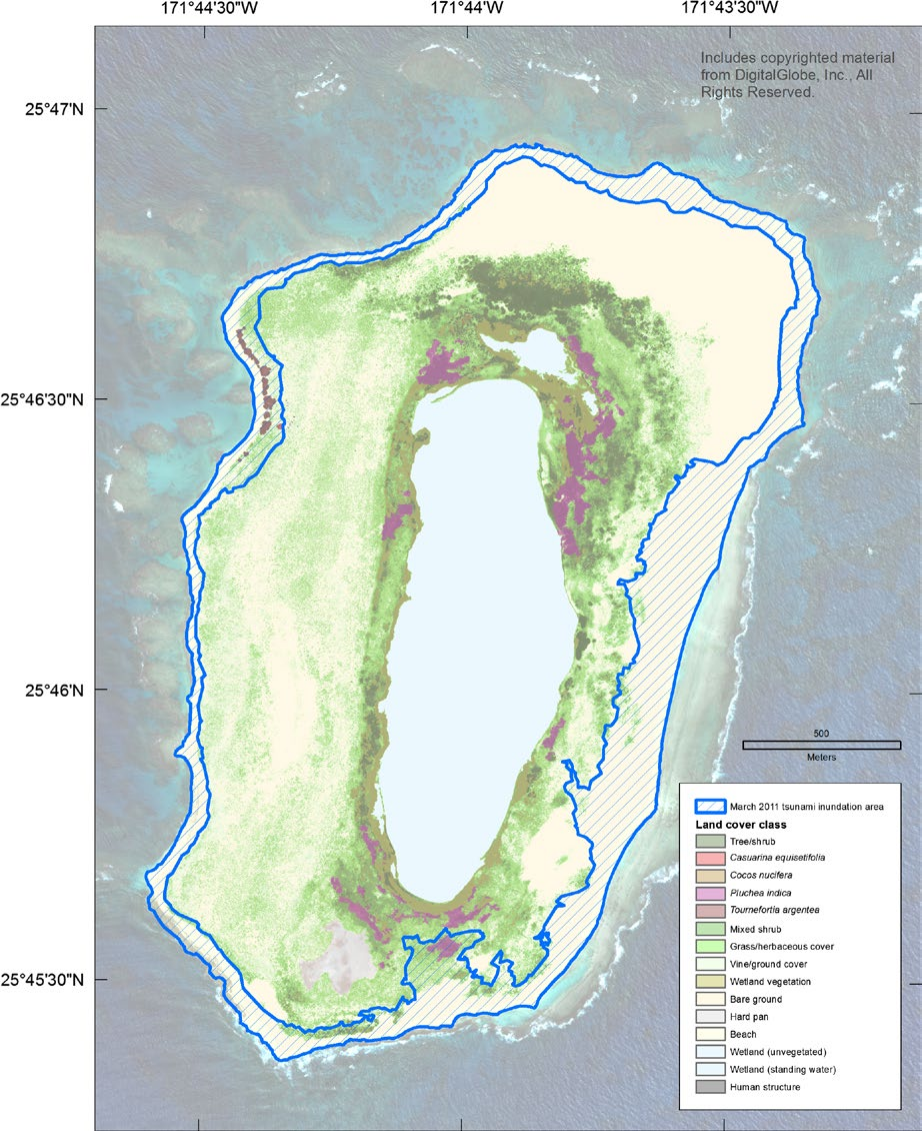
Inundation of land cover at Laysan Island, Hawai'i during the March 2011 Tōhoku tsunami

The tide station at Sand Island, Midway Atoll recorded eight events with water heights of at least 30 cm from 1952 through March 2017 (Table 3; NOAA 2017). On 11 March 2011, a peak water level of 1.6 m was recorded at 10:48 UTC (23:48, 10 March 2011 local time [Samoa Standard Time]), 5:02 hr after the Tōhoku earthquake. The tsunami traveled 3,876 km to Midway Atoll at an average speed of over 770 km/hr (NOAA 2011, 2015, 2017). The largest wave at Laysan Island was reported to have arrived at approximately 12:30 UTC on 11 March 2011 (02:30 local time [Hawai'i Standard Time]; USFWS unpublished data). The maximum terrestrial elevation (or wave run‐up elevation) flooded was 7.9 m at Sand Island, 2.6 m at Spit Island, 5.6 m at Eastern Island, and 7.7 m at Laysan Island (Table 4). The maximum wave run‐up distance from the coastline was approximately 500 m at Sand Island, 500–800 m at Eastern Island, and 300 m at Laysan Island; Spit Island experienced complete overwash (approximately 300 m; Table 4, Figures 4 and 5). Based on each island's inundation line (including the maximum range of GPS error), the extent of flooding was 41% (range: 39%–42%) at Midway Atoll with 29% inundation at Sand Island, 100% at Spit Island, 78% at Eastern Island, and 21% (of terrestrial area, excluding the central lake; range: 20%–23%) at Laysan Island (Table 4, Figures 4 and 5).

**Table 3:** Tsunamis recorded at Midway Atoll and Laysan Island, Hawai'i (1896, 1933, 1952–2016) with maximum tide gauge readings of ≥0.3 m above MSL at Sand Island, Midway Atoll

Date (year‐month‐day)	Earthquake Source	Earthquake magnitude (Mw)^1^	Maximum water height (m) at Sand Island	Impacts/damage at Midway Atoll	Impacts/damage at Laysan Island
1896‐06‐15	Sanriku, Japan	8.3	Not measured (predates tide gauge)	Unknown^1,2,3^	Evidence of inundation noted but not detailed^4^
1933‐03‐02	Sanriku, Japan	8.4	Not measured (predates tide gauge)	Notable tsunami waves observed^2^	Unknown^1,2,3^
1952‐11‐04	Kamchatka Peninsula, Russia	9.0	1.90	Sand and debris deposited hundreds of meters inland^3^	Unknown^1,2,3^
1957‐03‐09	Aleutian Islands, Alaska, USA	8.6	0.41	Unusual flooding^2^; many young albatross washed away or drowned^5^	Unknown^1,2,3^
1960‐05‐22	Valdivia, Chile	9.5	0.60	Unknown^1,2,3^	Unknown^1,2,3^
1963‐10‐13	Kuril Islands, Russia	8.5	0.30	Unknown^1,2,3^	Unknown^1,2,3^
1986‐05‐07	Aleutian Islands, Alaska, USA	8.0	0.34	Unknown^1,2,3^	Unknown^1,2,3^
2006‐11‐15	Kuril Islands, Russia	8.3	0.47	None; surfline approximately 1 m higher than expected^6^	None; abnormal waves and coral reef exposed^7^
2010‐02‐27	Maule, Chile	8.8	0.32	None^6^	None; abnormal waves and coral reef exposed^8^
2011‐03‐11	Tōhoku, Japan	9.0	1.57	Limited infrastructure effects^1,6,9^; numerous wildlife and vegetation impacts^6,9^	Numerous wildlife and vegetation impacts^10,11^

Data sources: 1: NOAA (2017); 2: Pararas‐Carayannis and Calebaugh (1977); 3: Lander and Lockridge (1989); 4: Schauinsland (1899); 5: Rice (1959); 6: JLK personal observation; 7: Murdoff, Freeman, and Metheny (2007); 8: Kristof et al. (2010); 9: O'Brian (2015); 10: Kristof, Watson, Cook, and Tyhurst (2011); 11: A. Kristof, USFWS, personal communication.

**Table 4:** Flooding of Midway Atoll and Laysan Island, Hawai'i during the March 2011 Tōhoku tsunami. Laysan Island values are excluding and including the interior lake. Flooding extent uncertainty is primarily a function of the accuracy of the mapped inundation line (<5 m error)

Location	Island area (ha)	Area inundated (ha)	Proportion inundated	Max. run‐up elevation (m)	Max. detectable run‐up distance (m)
Sand Island	457.7	132.0	0.29	7.9	~500
Spit Island	5.1	5.1	1.00	2.6	~300
Eastern Island	136.4	106.4	0.78	5.6	500–800
Midway Atoll total	599.2	243.5	0.41	7.9	500–800
Laysan Island terrestrial	337.8^a^	71.8	0.21	7.7	~300
Laysan Island total	412.0	71.8	0.17	7.7	~300

a This area excludes the large central hypersaline lake (74.2 ha)

Tsunami‐capable tide stations recorded maximum water heights of 2.0 m above the normal tide at Kahului, Hawai'i at 14:09 UTC and 2.5 m above the normal tide at Crescent City, California at 16:53 UTC (Dunbar, McCullough, Mungov, Varner, & Stroker, 2011; NOAA 2015). For Sand Island and Kahului, the largest recorded waves were the second waves in the event; on Sand Island, peak wave heights occurred approximately every 10 minutes, with the highest waves occurring in the first hour and then tapering off considerably after a few hours (http://tidesandcurrents.noaa.gov/tsunami/#; station ID 1619910).

### 3.2 Flooding of seabird nesting areas

**Figure 6:**
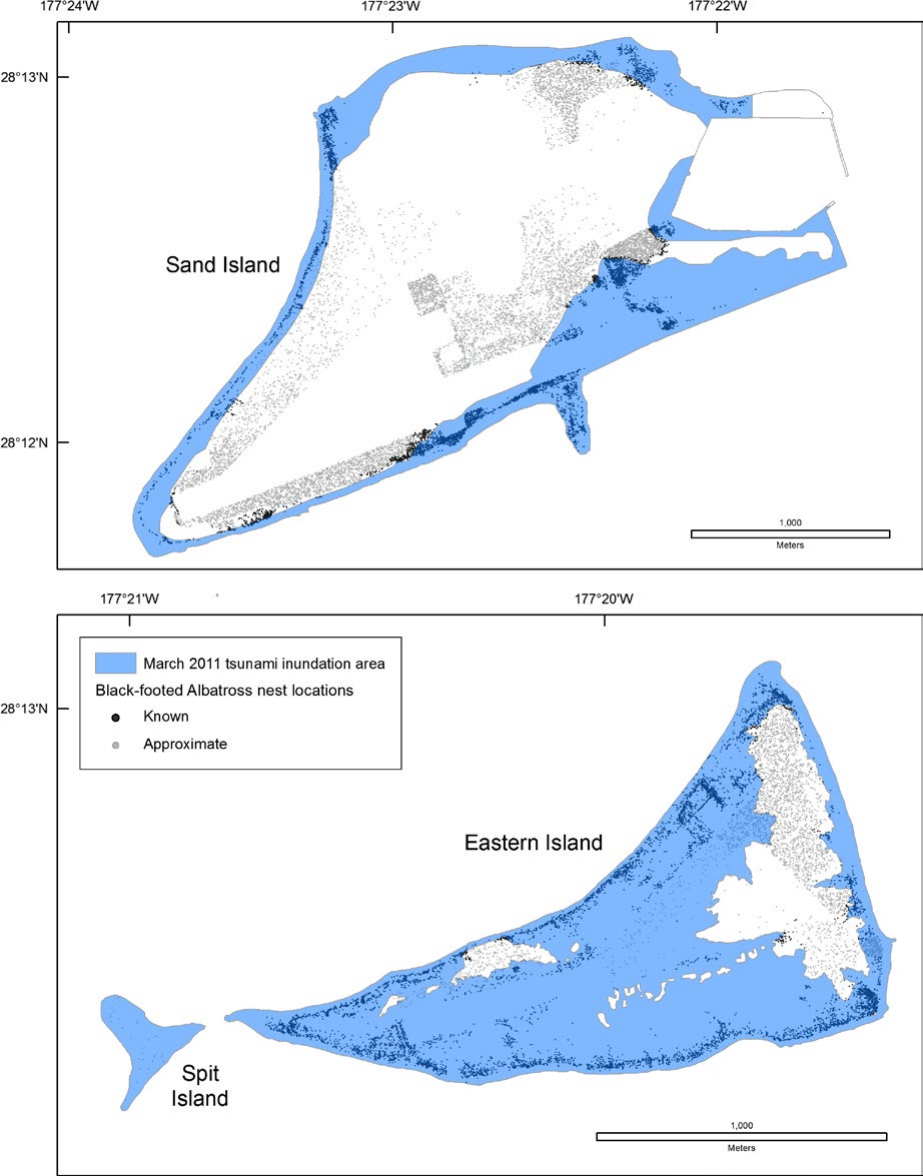
Overlay of the March 2011 Tōhoku tsunami inundation area and Black‐footed Albatross (*Phoebastria nigripes*) nests, Midway Atoll, Hawai'i

**Figure 7:**
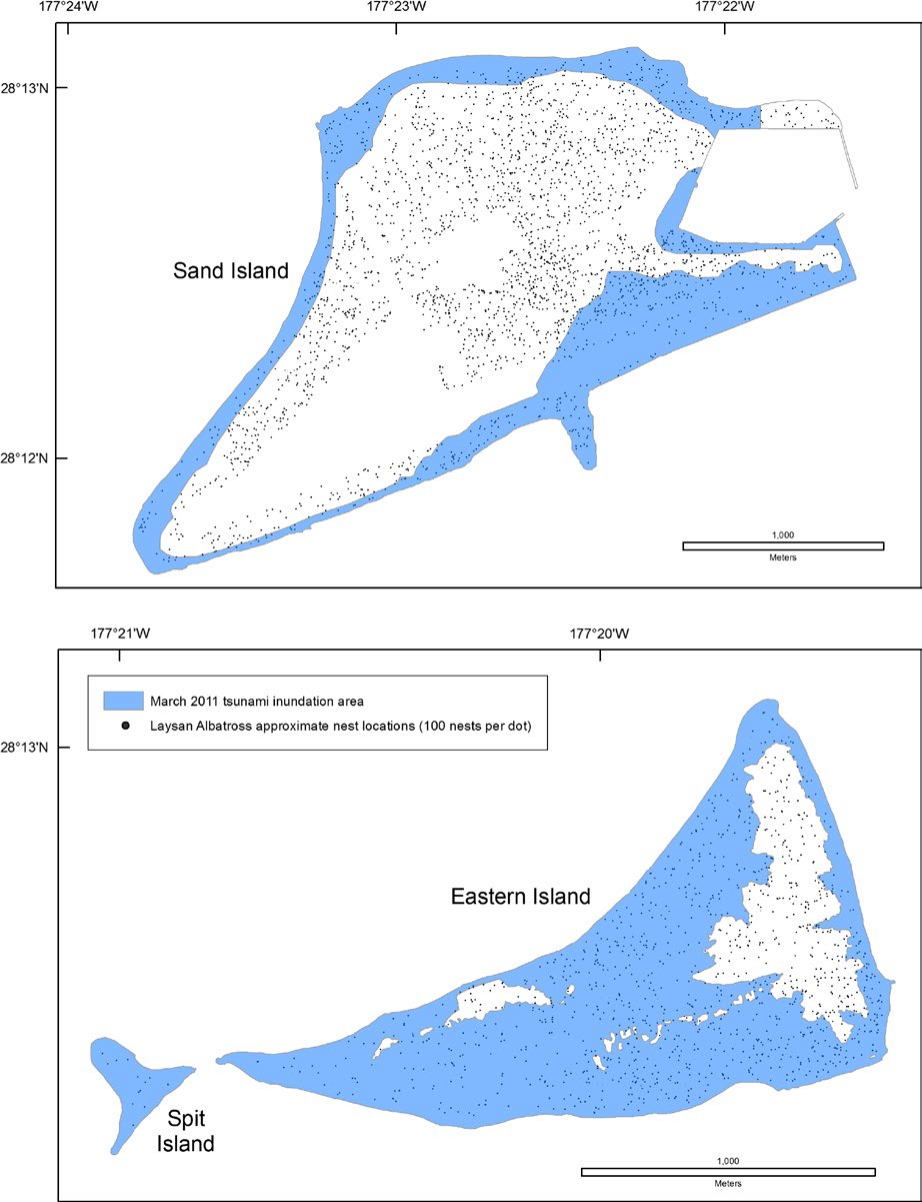
Overlay of the March 2011 Tōhoku tsunami inundation area and Laysan Albatross (*Phoebastria immutabilis*) nests, Midway Atoll, Hawai'i

**Figure 8:**
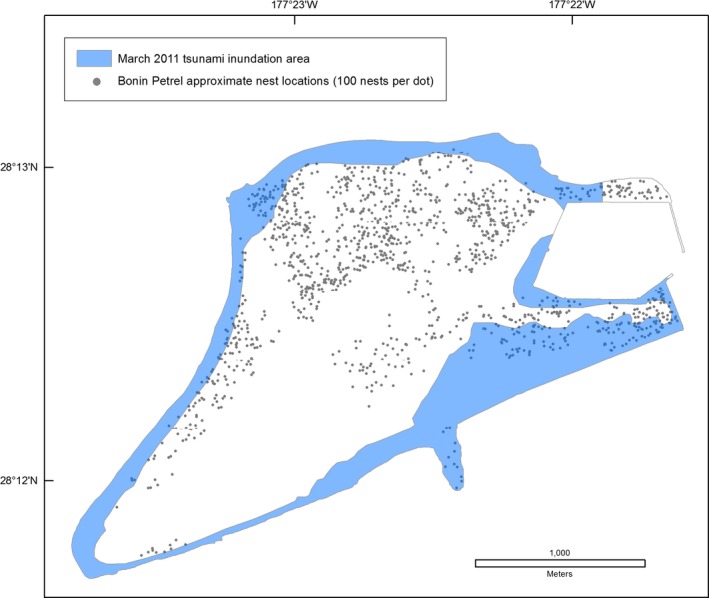
Overlay of the March 2011 Tōhoku tsunami inundation area and Bonin Petrel (*Pterodroma hypoleuca*) nesting area, Sand Island, Hawai'i

The Tōhoku tsunami struck Midway Atoll and Laysan Island during the night (02:30 local time), when many adult seabirds are on land to attend their nests or roost. The sudden flooding coincided with the breeding season of 14 of 23 species. At Midway Atoll, tsunami inundation overlapped 52% of Black‐footed Albatross nests, 45% of Laysan Albatross nests, the Short‐tailed Albatross nest and social attraction area, and approximately 20% of Bonin Petrel nests (Table 5, Figures 6, 7, 8). All albatross nests at Spit Island were flooded. Black‐footed and Laysan albatross nests at Eastern Island experienced 75%–79% inundation and the only Short‐tailed Albatross nest was flooded (Table 5). On all islands, Black‐footed albatross nests were concentrated in coastal areas (Figure 6), while Laysan Albatross and Bonin Petrel nests were distributed across the atoll (Figures 7 and 8; also see Reynolds, Courtot, Berkowitz et al., 2015).

**Table 5:** Nest flooding estimated at Midway Atoll and Laysan Island, Hawai'i during the March 2011 Tōhoku tsunami for species with nest distribution and population abundance data (Reynolds et al., 2012; Reynolds, Courtot, Berkowitz et al., 2015). See Table 2 for methods

Species	Island	No. of nests	Projected no. of nests inundated	Projected proportion of nests inundated
Black‐footed Albatross (*Phoebastria nigripes*)	Sand	15,002	5,351	0.36
	Spit	28	28	1.00
	Eastern	10,413	7,800	0.75
	Midway Atoll total	25,443	13,179	0.52
	Laysan	22,272	5,791	0.26
Laysan Albatross (*Phoebastria immutabilis*)	Sand	288,409	65,713	0.23
	Spit	1,498	1,498	1.00
	Eastern	193,002	152,420	0.79
	Midway Atoll total	482,909	219,631	0.45
	Laysan	115,166 ± 23,338	19,578 ± 3,967	0.17
Bonin Petrel (*Pterodroma hypoleuca*)	Sand	129,534	25,837	0.20
Masked Booby (*Sula dactylatra)*	Laysan	163	16	0.10
Brown Booby (*Sula leucogaster*)	Laysan	35	11	0.31

±, 95% confidence interval.

**Figure 9:**
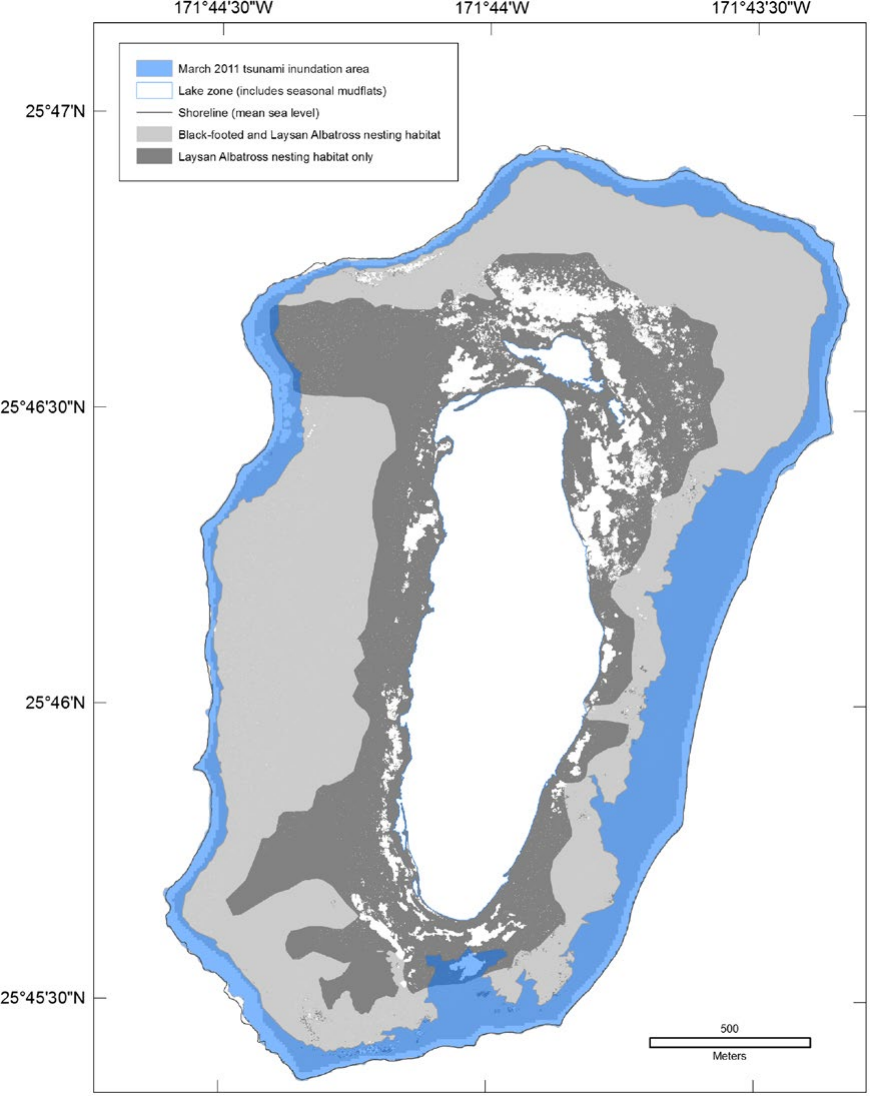
Overlay of the March 2011 Tōhoku tsunami inundation area and Black‐footed (*Phoebastria nigripes*) and Laysan albatross (*P. immutabilis*) nesting areas, Laysan Island, Hawai'i

At Laysan Island, the tsunami flooding covered 26% of Black‐footed and 17% of Laysan Albatross nesting habitat (Figure 9). Ten percent of Masked and 31% of Brown Booby nests that were mapped were flooded (Table 5). Eleven percent of the nesting area used by Red‐footed Boobies and Great Frigatebirds was inundated (Table 6).

**Table 6:** Projected habitat inundation at Midway Atoll and Laysan Island, Hawai'i during the March 2011 Tōhoku tsunami for species with distribution or habitat use data (Reynolds et al., 2012; Reynolds, Courtot, Berkowitz et al., 2015)

Species	Island	Habitat type	Habitat area (ha)	Projected habitat area inundated (ha)	Projected proportion of habitat area inundated
Bonin Petrel (*Pterodroma hypoleuca*)	Eastern	Nesting	1.55	1.52	0.98
Red‐footed Booby (*Sula sula*) and Great Frigatebird (*Fregata minor*)	Laysan	Nesting	14.8	1.6	0.11
Laysan Teal (*Anas laysanensis*)	Midway Atoll	Nesting and foraging	366.4	150.0	0.41
Laysan Teal and Laysan Finch (*Telespiza cantans*)	Laysan	Nesting and foraging	172.0	12.2	0.07

Nesting habitat inundation at Midway Atoll reached 49% of grass/herbaceous cover (e.g., *Lobularia maritima, Eragrostis variabilis,* V*erbesina encelioides*) and 57% of bare ground (Tables 1 and 7). At Midway Atoll, 24% of *Casuarina equisetifolia* trees and 45% of the other tree/shrub habitat (e.g., *Scaevola taccada*,* Tournefortia argentea*) was flooded (Table 7). At Laysan, most of the suitable nesting habitat had <10% inundation except for bare ground, which experienced 31% inundation (Table 7). The peak of the Masked Booby (*Sula dactylatra*) nesting season coincided with tsunami inundation (Figure 3), and of their potential ground‐nesting habitat, 44% was inundated at Midway Atoll and 17% at Laysan Island (Tables 1 and 7). Similarly, tree and shrub canopy habitat used by White Terns (*Gygis alba*) was inundated at 33% at Midway Atoll and 5% at Laysan Island (Tables 1 and 7).

**Table 7:** Land cover at Midway Atoll and Laysan Island, Hawai'i classified and quantified from WorldView‐2 satellite imagery and area inundated by the March 2011 Tōhoku tsunami. Nesting habitat classified as: tree and shrub canopy (C), underneath trees, shrubs and dense bunch grasses on the ground (U), on the ground with vegetation or bare ground (G), and subterranean burrows and crevices (S). For more detailed descriptions of land cover classes, including species information, see Reynolds et al. (2012) and Reynolds, Courtot, Berkowitz et al. (2015)

Land cover class	Nesting habitat type	Midway Atoll^a^			Laysan Island^b^		
		Total area (ha)	Area inundated (ha)	Proportion inundated	Total area (ha)	Area inundated (ha)	Proportion inundated
Tree/shrub	C,U,S	56.6	25.5	0.45	12.3	0.1	0.01
*Casuarina equisetifolia*	C,U,G,S	84.9	20.7	0.24	0	0	1
*Cocos nucifera*	C,U,G,S	NQ	NQ	NQ	0	0	0
*Pluchea indica*	C,U	Ab	Ab	Ab	8.3	0.4	0.05
*Tournefortia argentea*	C,U	NQ	NQ	NQ	0.7	0.7	1
Mixed shrub	C,U,G,S	NQ	NQ	NQ	18	0.6	0.03
Grass/herbaceous cover	U,G,S	171.0	84.4	0.49	74.8	6	0.08
Vine/ground cover	G,S	53.8	19.4	0.36	58	4.3	0.07
Partially vegetated former runway	G	36.6	24.2	0.66	Ab	Ab	Ab
Wetland vegetation	G	NQ	NQ	NQ	13.8	0.3	0.02
Bare ground	G,S	43.3	24.5	0.57	129.3	40.5	0.31
Hard pan	G	Ab	Ab	Ab	3.1	0	0
Beach	Unsuitable	25.2	25.1	1	19.5	18.9	0.97
Wetland (unvegetated)	Unsuitable	NQ	NQ	NQ	34.2	0	0
Wetland (standing water)	Unsuitable	2.2	0.2	0.09	40	0	0
Human structures	Unsuitable	125.6	19.5	0.16	0	0	0
	Total	599.2	243.5	0.41	412	71.8	0.17

Ab, absent; NQ, not quantified, included in general land cover category.

a WorldView‐2 satellite imagery 14 January 2010 (DigitalGlobe Inc., 2010).

b WorldView‐2 satellite imagery 18 May 2010 (DigitalGlobe Inc., 2010).

### 3.3 Land bird population effects

**Figure 10:**
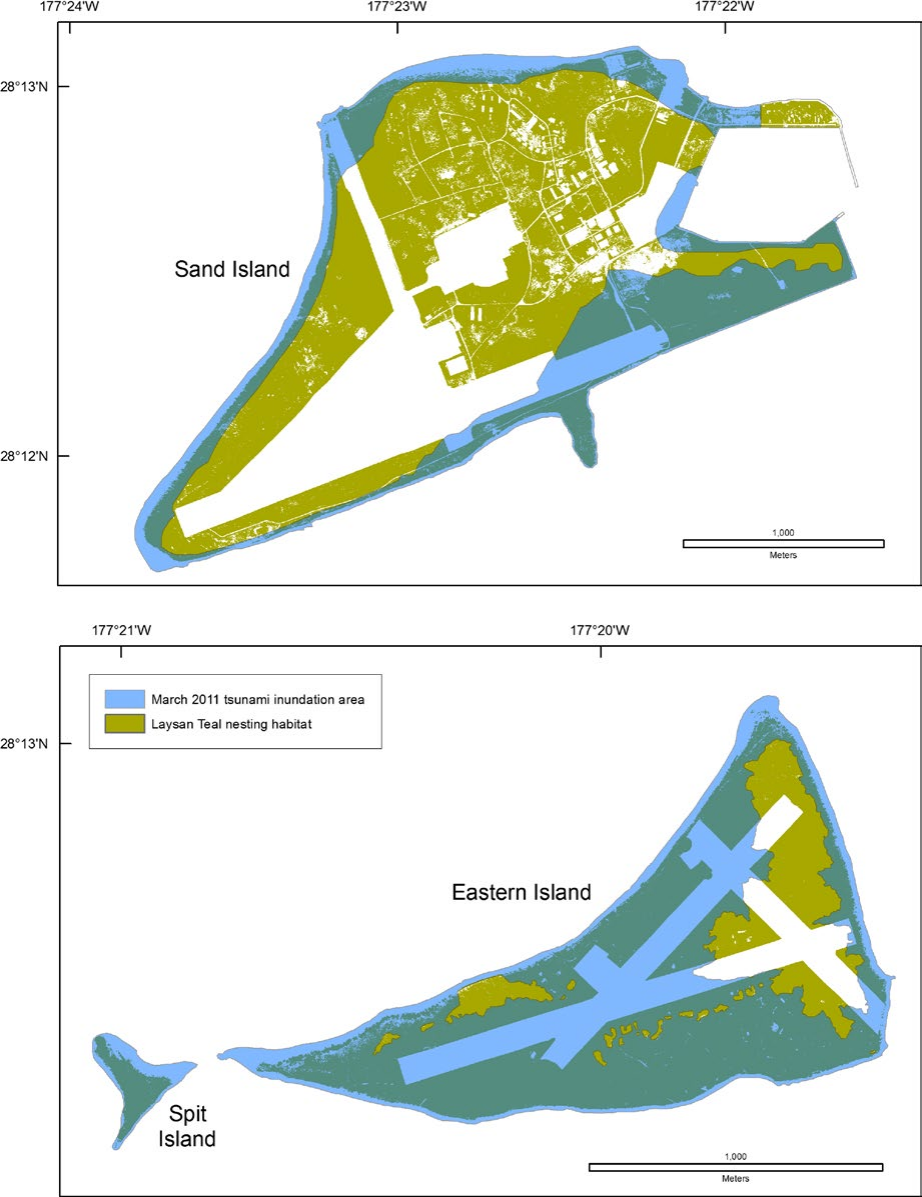
Overlay of the March 2011 Tōhoku tsunami inundation area and Laysan Teal (*Anas laysanensis*) nesting and foraging area, Midway Atoll, Hawai'i

**Figure 11:**
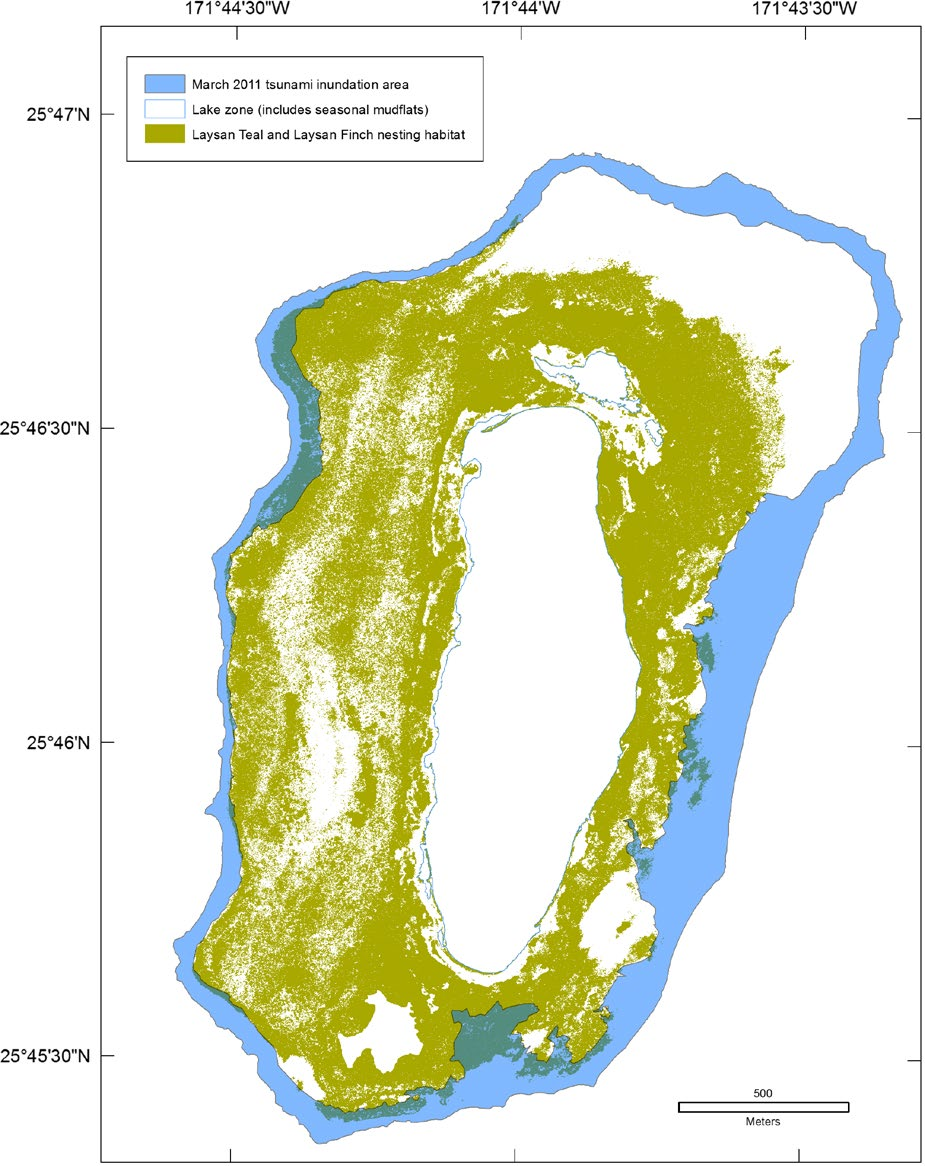
Overlay of the March 2011 Tōhoku tsunami inundation area and Laysan Teal (*Anas laysanensis*) and Laysan Finch (*Telespiza cantans*) nesting and foraging area, Laysan Island, Hawai'i

At Midway Atoll, 41% of Laysan Teal nesting and terrestrial foraging habitat was overwashed (Table 6, Figure 10). Six of 13 freshwater wetlands used by Laysan Teal and migratory waterbirds at Midway Atoll were overwashed and filled with tsunami debris, marine fish (e.g., *Acanthurus triostegus, Zebrasoma flavescens*), and green turtles (*Chelonia mydas*). Salinity was elevated from less than 0.01 g/100 g to >3.50 g/100 g for 2 months. Laysan Teal and Laysan Finch had 7% of their nesting and terrestrial foraging habitat overwashed at Laysan Island (Table 6, Figure 11).

**Figure 12:**
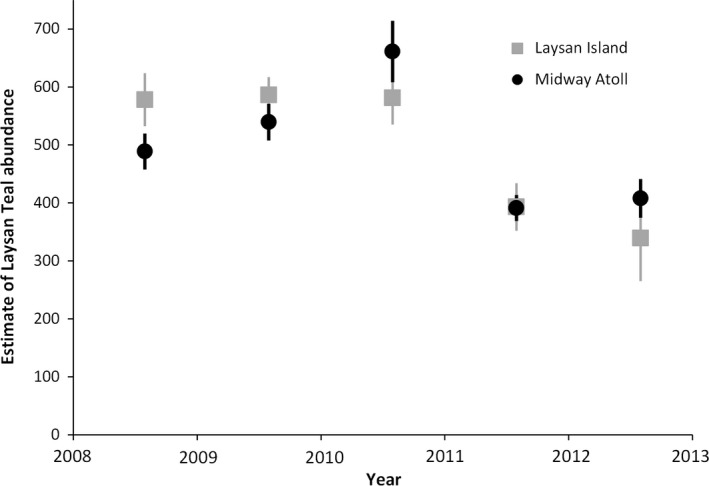
Modified Chapman bias‐corrected Lincoln‐Petersen estimates of Laysan Teal (*Anas laysanensis*) abundance at Midway Atoll and Laysan Island, Hawai'i, 2008–2012. Bars represent 95% confidence intervals

In addition to direct mortality of Laysan teal from sudden flooding, an outbreak of avian botulism (*Clostridium botulinum*) type C intoxicated Laysan Teal at Midway Atoll and coincided with the massive die‐off of seabirds after the tsunami (USFWS unpublished data; USGS National Wildlife Health Center, Honolulu, Hawai'i, unpublished data, 22 Mar–26 Sep 2011). Laysan Teal populations had reproductive failure in 2011 on Laysan and Eastern islands. Of the individually marked (leg banded) Laysan Teal, 22% at Midway Atoll and 18% at Laysan Island died within approximately 6 months of the tsunami. Following the tsunami, population abundance had declined 42% at Laysan Island (Reynolds, Courtot, Brinck et al., 2015) and 38% at Midway Atoll (USGS unpublished data; Figure 12).

## 4 DISCUSSION

### 4.1 Historical tsunamis and data limitations

Throughout the Pacific, major tsunamis have occurred approximately once per decade (NOAA 2015). Tsunami effects vary depending on the shape and depth of the source zone, wave directionality, and bathymetric and coastal characteristics of the affected run‐up location (NOAA 2017). From 1933 to 2016, approximately 50 run‐up events were documented at Midway Atoll; however, only two seismic events (November 1952 and March 2011) since tide gauge deployment recorded wave heights >1 m. The extent of flooding is not easily predictable based entirely on tide gauge data. In March 2011, the terrestrial elevations that were flooded greatly exceeded the maximum water levels recorded at tide gauges. Water levels at the tide gauges were 1.6 m above the normal tide (NOAA 2017) but water pushed inland to elevations of 7.9 m (i.e., run‐up). Our results highlight the difficulty estimating tsunami impacts and inundation extents based solely on tide gauge data.

Flood descriptions and damage reports on Pacific atolls are sparse. We found accounts but with few details for 10 tsunamis reaching the Northwestern Hawaiian Islands (Table 3). Reports from Midway Atoll describe a 1952 tsunami that deposited sand and debris on runways and moved buildings on Sand Island (Lander & Lockridge, 1989; NOAA, 2017). In 1957, tsunami waves washed away and drowned albatross chicks (Pararas‐Carayannis & Calebaugh, 1977; Rice, 1959). Although damage to infrastructure at Midway Atoll from the Tōhoku tsunami was reported as “limited” by NOAA (2017), we estimated with our spatially explicit models that more than 40% of the atoll was inundated and damage to federally protected wildlife was extensive. Drowning and fatal entrapment of adults or chicks of eight species were observed (Figure 3; JLK personal observations, A. Kristof, USFWS, personal communication).

Other remote islands of the Hawaiian archipelago have little information on past tsunamis because neither humans nor tide gauges were present. A naturalist that visited Laysan Island in June 1896 noted evidence of inundation from a tsunami generated by an earthquake in Japan, but the extent of inundation and effects to wildlife remain unknown (Schauinsland, 1899). Nearby Kure Atoll reported approximately 2,200 Black‐footed Albatross and 2,000 Laysan Albatross chicks lost during the 2011 Tōhoku tsunami (Hawaii Dept. of Land and Natural Resources, unpublished data). Inundation of Pearl and Hermes Atoll (Figure 2) appeared to approach 30%–50% of the islands based on photographs taken during an overflight on 22 March 2011 (USFWS unpublished images); however, the extent of flooding appeared limited at Lisianski Island (USFWS unpublished images) and the islands of French Frigate Shoals (P. Hartzell USFWS, personal communication).

During 2017, bird populations were monitored on only four of the 22 Northwestern Hawaiian Islands, with just five of 23 species being monitored regularly for abundance. As population monitoring data are lacking for most species, potential nest losses and population changes from future overwash events are likely to be unknown. On isolated islands, remote sensing and the use of unmanned aircraft systems (UAS) are potential tools to make up for limited population data for many species and to record inundation extents from sudden flooding events (also see, Christie, Gilbert, Brown, Hatfield, & Hanson, 2016).

### 4.2 Other studies of tsunami impacts on wildlife

Few studies have documented the impacts of tsunamis on wildlife populations. After the December 2004 Indian Ocean tsunami following the Sumatra‐Andaman earthquake, short‐term effects (e.g., Sivakumar, 2009; Viera et al., 2006) and habitat changes were described (e.g., Hayasaka, , Goka et al., 2012; Hayasaka, Shimada et al., 2012; Kendall et al., 2009; Kumar, Chingkhei, & Dolendro, 2007). Following the 2010 Chilean tsunami, massive losses of Cabbage Trees (*Dendroseris litoralis*), an important seasonal nectar supply for the critically endangered Juan Fernandez Firecrown (*Sephanoides fernandensis*), led to short‐term changes in hummingbird distribution and abundance. This in turn may have resulted in population declines over the long term (Hahn et al., 2015). Future studies of ecosystem response to sudden flooding are needed to understand how these catastrophes influence island biodiversity and how sea‐level rise interacts with other stressors.

### 4.3 Coastal vegetation impact

**Figure 13:**
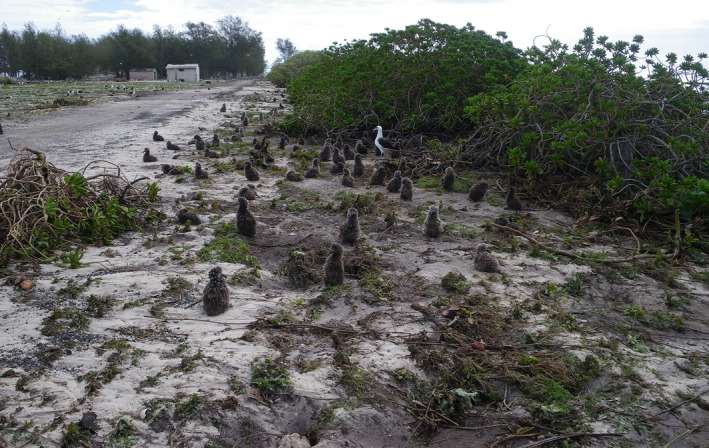
Coastal vegetation physically uprooted and albatross chicks washed from their nests by waves at Midway Atoll, Hawai'i during the March 2011 Tōhoku tsunami

The estimated speed of waves washing over land at Midway Atoll was 27.7 km/hr (R. Weiss, Virginia Tech, personal communication). Coastal vegetation was physically uprooted by waves (Figure 13), and trees and shrubs in flooded areas died due to salt water exposure. Vegetation cover, structure, and composition were affected, especially for invasive *V. encelioides* that was reduced by the initial overwash at Midway Atoll. However, within a year of the disturbance, the resultant competitive release benefitted *V. encelioides* (JLK, personal observations). *Scaevola* spp. may protect against erosion, trap floating debris, and diminish wave impact (Collen, Garton, & Gardner, 2009; Sundaresan, 1993) and indeed, at Midway Atoll during this event, dense stands of *Scaevola taccada* were associated with protective dunes and appeared less affected than other areas (JLK, personal observations).

### 4.4 Vulnerability of bird communities

Using the Tōhoku tsunami as a case study, we provided a model to show the variation in vulnerability and impact to island biodiversity from sudden inundation events. This study illustrated how vulnerable low‐lying islands are to sudden flooding. Population vulnerability to catastrophic events varies with differences in spatial and temporal exposure and sensitivity to the effects of the event (e.g., Gardali, Seavy, DiGaudio, & Comrack, 2012; Reynolds, Courtot, Berkowitz et al., 2015). Exposure to inundation is greater for species that concentrate near the coastline (e.g., Brown Booby and Black‐footed Albatross), compared to species that typically nest farther inland (e.g., Red‐footed Booby, Great Frigatebird), or species with nest distributions across the island (e.g., Laysan Finch). Ground‐nesting species such as albatrosses and Gray‐backed and Sooty terns may suffer direct mortality from sudden flooding, or chicks displaced from their nests may not survive to independence. Tree‐ and shrub‐nesting species (e.g., White Tern) may be less exposed to sudden flooding, unless trees are uprooted or damaged by waves or debris.

During the month of March when the tsunami struck, adults, chicks, eggs, and entire colonies of 14 species were vulnerable to sudden flooding. By contrast, due to seasonal differences in species presence and/or investment in reproduction, a similar inundation event in October would coincide with the peak nesting season of only two species. The mortality of adult breeders from sudden flooding has disproportionate effects on some species for which the mortality of one parent typically results in chick or egg mortality, as well as mate loss and reduced future reproductive potential. Albatrosses, petrels, boobies, and frigatebirds are long‐lived seabirds with deferred maturity, low fecundity, and high adult survival (Nelson, 1978; Warham, 1996; Weimerskirch, 2001). In contrast, species that lay replacement eggs or have asynchronous or aseasonal breeding (e.g., Black Noddy, White Tern) will likely be less sensitive to sudden flooding during their nesting season compared with species limited to a single nesting attempt or nest synchronously (e.g., albatrosses and petrels; Reynolds, Courtot, Berkowitz et al., 2015).

As sea level rises, insular wildlife will be increasingly exposed to flooding (Baker, Littnan, & Johnston, 2006; Bellard, Leclerc, & Courchamp, 2014; Reynolds et al., 2012). Higher sea levels in the coming decades potentially will cause greater inundation extents, increased flooding depths and durations, and increased flooding frequency (Eversole & Andrews, 2014), all of which have the potential to cause declines in bird populations and colony collapses.

### 4.5 Conservation conclusions

Range‐restricted and island species are vulnerable to extinction (Jenkins, Van Houtan, Pimm, & Sexton, 2015), and our spatially explicit models of tsunami inundation highlight the exposure of species nesting on Pacific islands. On the four islands of our study, a range of up to 6–10 million birds may rely on a combined area of about 9.3 km^2^, with a mean elevation of less than 3.5 m (Reynolds et al., 2012). Midway Atoll and Laysan Island support the largest colonies of Black‐footed and Laysan albatrosses globally and, in total, more than 95% of the global populations of these strongly philopatric albatrosses nest on the low‐lying Northwestern Hawaiian Islands (Arata, Sievert, & Naughton, 2009; Fefer, Harrison, & Naughton, 1984). These islands also support the global populations of two resident land bird species: endangered Laysan Finch and Laysan Teal. Additionally, the endangered Nihoa Millerbird (*Acrocephelus familiaris kingi*) was translocated to Laysan Island after the Tōhoku tsunami (Freifeld et al., 2016). Long‐term population‐level effects from infrequent catastrophic sudden flooding are unlikely for most long‐lived seabirds (Weimerskirch, 2001), especially where dispersal to similar alternative habitat is available. However, island biodiversity and population resilience is lost when catastrophes are combined with other anthropogenic threats including invasive predators and climate change (Blackburn, Cassey, Duncan, Evans, & Gaston, 2004). Pacific island avifauna is especially vulnerable to the loss of predator‐free nesting habitat as this prevents opportunities for successful dispersal, immigration, and breeding. The frequency of catastrophic sudden flooding on low‐lying Pacific islands may increase with sea‐level rise combined with storm wave run‐up. The value of restoration of higher elevation habitat for recolonization by Pacific birds is evident, particularly for species with ranges currently restricted to low‐elevation sites susceptible to tsunamis.

Digital elevation models, spatial vulnerability mapping, and species sensitivity analyses are important tools to help evaluate proposed restoration and conservation actions. Exposure to sudden flooding could be used in the decision‐making process to evaluate the suitability of habitat restoration or social attraction sites (techniques using decoys and vocalization recordings to lure colonially nesting birds; Kress, 1983; Young & VanderWerf, 2016). Digital elevation models, mapping from the Tōhoku tsunami, tsunami hazards forecasting, and projections of storm wave inundation and sea‐level rise (Gica, 2015; Hatfield, Reynolds, Seavy, & Krause, 2012; Reynolds et al., 2012; Reynolds, Courtot, Berkowitz et al., 2015) reveal the zones and islands that are the most vulnerable to sudden flooding. This information could inform translocation and social attraction conservation actions. For example, Black‐footed Albatross chicks translocated from Midway Atoll to establish future breeding colonies at higher elevation areas protected by predator‐proof fences (USFWS 2017) could be selected from nests on Spit and areas of Eastern Islands that are vulnerable to increasing risk of sudden flooding with sea‐level rise. Additionally, the conservation value of attracting long‐lived endangered Short‐tailed Albatrosses to Eastern Island of Midway Atoll, far from foraging and other breeding sites (Suryan et al., 2006, 2008), warrants review in light of new scenarios of sea‐level rise (Reynolds, Courtot, Berkowitz et al., 2015) and tsunami forecasts at Midway Atoll (Gica, 2015). Reintroduction of other endangered taxa (e.g., Laysan Finch, Laysan Teal, Nihoa Finch (*Telespiza ultima*)), endangered plants, or Hawaii green turtles to additional islands could be considered a useful short‐term or intermediate step in a conservation strategy to reduce extinction risks, or as an alternative to captivity, until a longer‐term strategy to restore larger and higher elevation sites for recolonization (via translocation, social attraction, or natural immigration) can be implemented.

## CONFLICT OF INTEREST

None declared.

## AUTHOR CONTRIBUTIONS

M.H.R and K.N.C. conceived and designed the study; J.L.K and K.N.C collected field data; P.B. and K.N.C. analyzed data; all authors contributed to writing the final manuscript.
